# Scoping review of implementing a longitudinal curriculum in undergraduate medical education: The wake forest experience

**DOI:** 10.1186/s13089-021-00206-w

**Published:** 2021-04-19

**Authors:** Casey Glass, Aarti Sarwal, Joshua Zavitz, Joshua Nitsche, JaNae Joyner, Leilani L. Johnson, Julia Garcia-Vargas, Mary Claire O’Brien

**Affiliations:** 1 Department of Emergency Medicine, Medical Center, Wake Forest School of Medicine, 1 Winston-Salem, Boulevard, NC 27157 USA; 2 Department of Neurology, Medical Center, Wake Forest School of Medicine, 1 Winston-Salem, Boulevard, NC 27157 USA; 3 Department of Obstetrics and Gynecology, Wake Forest School of Medicine, Medical Center, 1 Winston-Salem, Boulevard, NC 27157 USA; 4 Department of Medical Education, Wake Forest School of Medicine, Medical Center , 1 Winston-Salem, Boulevard, NC 27157 USA

**Keywords:** Medical education, Ultrasonography, Undergraduate medical education, Ultrasound, Curriculum

## Abstract

**Background:**

Hands-on ultrasound experience has become a desirable component for undergraduate medical education (UGME) curricula throughout medical schools in the United States (US) to enhance readiness for future training. Ultrasound integration can be a useful assistive educational method in undergraduate medical education to improve anatomy and physiology skills. Relatively few medical schools have integrated ultrasound experiences formally into their 4-year medical school curriculum due to limitations of a resource intensive set up.

**Methods:**

We undertook a scoping review of published UGME ultrasound curricula integrated into all four years in peer-reviewed as well online literature. In addition, we provide a narrative review of our institutional experience in conceptualization, design and implementation of UGME ultrasound curriculum driven by need to address the fading knowledge in anatomy and physiology concepts beyond pre-clinical years.

**Results:**

Integrated ultrasound curriculum at WFSOM utilizes focused ultrasonography as a teaching aid for students to gain a more thorough understanding of basic and clinical science concepts taught in the medical school curriculum. We found 18 medical schools with ultrasound curricula published in peer-reviewed literature with a total of 33 ultrasound programs discovered by adding Google search and personal communication

**Conclusions:**

The results of the review and our institutional experience can help inform future educators interested in developing similar curricula in their undergraduate programs. Common standards, milestones and standardized competency-based assessments would be helpful in more widespread application of ultrasound in UGME curricula.

## Introduction

The integration of hands-on ultrasound experience has become a highly desirable component for undergraduate medical education (UGME) curricula throughout the United States (US) as part of enhanced efforts to improve readiness of future doctors. Several schools have implemented a student-performed ultrasound experience to a variable degree throughout the formal 4-year medical school program and many individual medical specialties have incorporated ultrasound into their electives highlighting ultrasound based clinical applications [1, 2]. Programs have described the impact of ultrasound integration in medical education curriculum in helping enhance traditional learning of anatomy, medical physiology, and clinical skills of their medical students.

We investigated the published literature on curricular integration and its structure at a medical school level inform on factors to consider during UGME program development concerning ultrasound integration. We provide a scoping review of published curricular key metrics necessary for the development and implementation of UGME ultrasound program. We then describe our institutional experience in integrating ultrasound into the Wake Forest School of Medicine UGME curriculum emphasizing the challenges and lessons learned.

This synopsis on challenges, successes and impact in integrating ultrasound into UGME pre-clinical and clinical years can hopefully inform future UGME program development for schools exploring ways to improve their undergraduate programs.

## Methods

1. A scoping review of literature was performed by authors (LJ, JGV, AS) using the electronic database PubMed, MEDLINE and the Cochrane Library in English literature using the following search words “Ultrasonography”, Ultrasound”, with the Boolean operators AND “Medical education”, “Undergraduate”, “School”, “clerkship” AND “Curriculum” [2]. Two authors (LJ and JGV) independently manually reviewed titles and abstracts resulting in a list of specific articles that met the criteria of describing an UGME ultrasound curriculum program development. Search methods conform to the Preferred Reporting Items for Systematic Reviews and Meta-Analyses (PRISMA) guidelines for scoping reviews [3]. These were reviewed for full text to describe key features of each ultrasound curricula (LJ, JGV, AS). Since the emphasis of this review was to assess school of medicine supported curricula, articles were only included if they described the integrated ultrasound curriculum in relevance to UGME structure. Articles that restricted curriculum information to one specialty only were not included. This data was supplemented by open internet search through Google search engine for other curricula using the search words: “Ultrasonography”, Ultrasound”, with the Boolean operators AND “Medical education”, “Undergraduate”, “School”, “clerkship” AND “Curriculum”. The results found were manually reviewed by authors (JGV and LJ) to look for publications that described full curricula. The first 10 pages of the Google results were manually reviewed to look for other published curricula. One of the authors (JGV) used survey-based questions via personal communication to complete the requisite information on unpublished ultrasound curricula to provide a comprehensive review of available programs (Table 1 and Appendix 1: Table 6). Questions were designed to address common components and themes in the curricula that were frequently discussed in published ultrasound curricula identified through literature review.
2. A narrative review of conceptualization, design, implementation and integration of ultrasound curriculum at WFSOM is presented emphasizing challenges and innovation. We describe the evolution of this curriculum based on framework provided by Kern [4].

**Table 1:** Chronology of ultrasound curriculum development at wake forest school of medicine

2016	2017	2018	2019
Introduction to Ultrasound Cardiac Anatomy Abdomen and Pelvis Anatomy Neck Anatomy Neurovascular Ultrasound Cardiac Function 1 and 2 Hepato-Biliary Ultrasound Renal System Ultrasound	Musculoskeletal Ultrasound Endocrine Ultrasound Ultrasound Guided Vascular Access Ultrasound for Pregnancy Point-of-Care Ultrasound elective	Lung Ultrasound Point-of-Care Echocardiography Dedicated teaching sonographer added to curriculum	Ultrasound certificate program Ultrasound simulator

## Results

### Scoping review of ultrasound curricula in the US

**Fig. 1:**
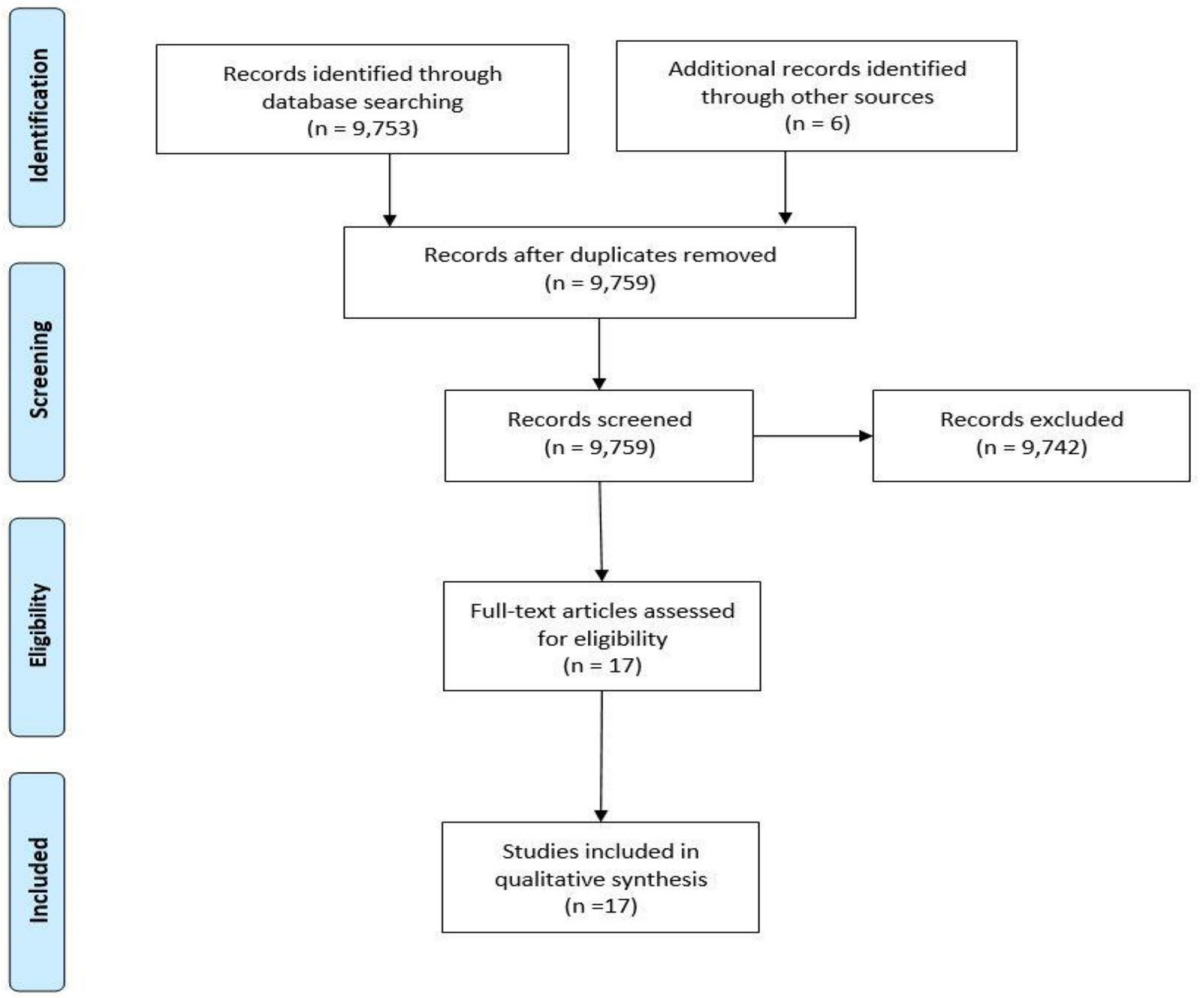
PRISMA diagram showing scoping review employed by authors

A scoping literature review of published ultrasound curricula was done to assess incorporation of ultrasound education in undergraduate medical education programs. This search yielded a total of 9,753 results. The review of titles and abstracts narrowed the results and full texts were reviewed by two independent authors (LJ and JGV). Design, implementation, and assessment of ultrasound education were described at 18 different institutions. Six additional records were included subsequently, four of these were personal references of senior authors and two were secondary references found on full text review of initial search (Fig. 1). A review of common data elements for these programs in presented in Table 2.

**Table 2:** A scoping review of various ultrasound curricula of undergraduate medical education reported in published literature

Institution	Reference	Duration (years)	Format of information delivery	Instructional method	Curricular structure	Mode of Integration	Preclinical years	Clinical years	Instructors	Student-to-Instructor Ratio
Touro University College of Osteopathic Medicine	Hendriksz, Markman, and Pera [27]	4	Hands-on sessions	Not addressed	Staged/ Organ-systems based	Concurrently with anatomy and clinical courses	Anatomy system blocks Cardiac and blood vessel Liver, gallbladder, and kidneys Ocular Fetal FAST examination Cardiac, vasculature, renal, lung	Small-group hands-on learning sessions Expanded radiology	Faculty	8: 1
University of California, Los Angeles University of California, Davis University of California, San Francisco University of California, San Diego University of Southern California Stanford University Loma Linda University	Chiem et al. [10]	4	Online materials (lectures, textbook chapters, simulated cases, and journal articles) + hands-on sessions	Flipped classroom	Staged/Organ-systems based	Concurrently with anatomy and physical examination courses	Ultrasound Image Acquisition of Normal Anatomy and Physiology Basic Ultrasound Pathology Ultrasound Identification of Pathologic Conditions	Pre-Senior Year Course Clerkships	Peer educators	Not addressed
University of California, Irvine	Fox et al. [28] Wilson et al. [29]	4	Open-access podcast lectures + hands-on sessions	Not addressed	Staged/Organ-system based	Expanded medical program of study	Knobology Cardiovascular 1 and 2 GI Physiology Respiratory Musculoskeletal Genitourinary Head and Neck Review/Practice Ultrasound for Evaluation of Fever Focused Assessment of the Thorax Exam (FATE) Lung Ultrasound Gastrointestinal and Genitourinary Ultrasound Cumulative Skills	Two-hour workshop for identification of pathology prior to rotations	Peer educators with faculty assistance	4: 1
Wayne State University School of Medicine	Rao et al. [12]	1	Didactics, hands-on experience, clinical correlation components	Flipped classroom	Vertical	Expanded medical program of study	Introduction to Ultrasound Musculoskeletal Ultrasound Vascular and Cardiac Ultrasound Ultrasound of the Abdomen Genitourinary Ultrasound Ultrasound and Procedural Skills	Not applicable	Peer educators with faculty assistance	Not addressed
University of South Carolina Medical School	Hoppmann et al. [4] Hoppmann et al. [1]	4	Web-based learning modules, video assignments, hands-on lab session	Not addressed	Vertical	Concurrently with anatomy, problem-based learning, clinical skills	Anatomy Physiology Neuroanatomy Problem-Based Learning Introduction to clinical medicine: pathophysiology Pathology Physical diagnosis	Emergency medicine "selective" Emergency medicine elective Critical care "selective" Radiology elective Clerkships (Internal Medicine, Family Medicine, Surgery, OB-GYN, Pediatrics) Ultrasound independent 1-month study elective Capstone ultrasound course selective Acting internship with ultrasound access	Faculty	Not addressed
West Virginia University School of Medicine	Mindari et al. [23]	4	Lectures, online videos, workshops, and practical hands-on sessions	Flipped classroom	Longitudinal	Concurrently as a "thread" with anatomy, basic science lectures, and physical examination courses	Human Structures (6 modules, including introduction to ultrasound) Microbiology and Immunology Pathology Pharmacology Physical Diagnosis/Clinical Integration	Clerkships (OB-GYN, Surgery, Internal Medicine, Family Medicine, Pediatrics, Psychiatry, Anesthesia, Critical Care) Emergency and Critical Ultrasound Elective Radiology Elective	Faculty	2: 1
Ohio State University College of Medicine	Bahner et al. [11] Bahner and Royall [7]	4	Lectures + hands-on sessions	Not addressed	Vertical + longitudinal	Concurrently with anatomy and clinical reasoning course	Musculoskeletal Anatomy Ultrasound Thorax, Abdomen and Pelvis Anatomy Ultrasound Head and Neck Anatomy Ultrasound Introduction to Focused Ultrasound Elective Basic Course in Focused Ultrasound Protocols Elective Ultrasound-Guided Vascular Access	Specialty-Based Hands-On Ultrasound Experience Core Focused Ultrasound Protocols Emergency Focused Ultrasound Specialty-Based Hands-On Ultrasound Experience	Faculty	Not addressed
Icahn School of Medicine at Mount Sinai	Nelson et al. [30]	1	Lectures, demonstration, hands-on session	Not addressed	Staged/Organ-systems based	Concurrently with gross anatomy and physical examination courses	Basic ultrasound physics and instrumentation Focused cardiac ultrasound Focused thoracic ultrasound Focused abdominal ultrasound	Not applicable	Faculty + senior residents	Not addressed
University of Sydney Medical School	Moscova et al. [31]	1	Hands-on sessions	Not addressed	Longitudinal	Incorporated into second year practical sessions	Imaging of abdomen, pelvis, and vascular ultrasounds	Note applicable	Specialists	Not addressed
A.T. Still University-Kirksville College of Osteopathic Medicine	Kondrashova and Lockwood [32] and Kondrashova [33]	2	Video modules, hands-on sessions, and clinical correlation components	Flipped classroom	Staged/Organ-systems based	Integrated into gross anatomy and new Clinical Ultrasound Elective	Introduction to US Neck Upper Limb Musculoskeletal Abdomen Gastrointestinal Pelvis Urinary system Lower Extremities Ocular Ultrasound Echocardiography Vascular & Extremities Clinical Ultrasound Elective	Not applicable	Not addressed	Not addressed
Rocky Vista University College of Osteopathic Medicine	Russ et al. [36]	4	Video modules, hands-on sessions, and clinical correlation components	Flipped classroom	Vertical	Integrated into gross anatomy and Principles of Clinical Courses	Ultrasound Physics and Introduction Musculoskeletal Parts I & II Cardiac Ultrasound Thoracic and Pulmonary Ultrasound Abdominal Ultrasound Head and Neck Ultrasound Cardiovascular Ultrasound Focused Abdominal Sonography in Trauma Ultrasound Abdominal Ultrasound Musculoskeletal Ultrasound Resuscitative Ultrasound	Ultrasonography and Procedure course Clinical Ultrasonography Rotation	Faculty + Peers	Not addressed
Harvard Medical School	Rempell et al. [15]	2	Lectures, case-based examples, hands-on sessions	Not addressed	Staged/organ-systems based	Integrated into gross anatomy and physical diagnosis course	Anatomy lab curriculum Ultrasound Selectives	Not applicable	Faculty	4: 1
Florida State University College of Medicine^*^	Not applicable	3	Large-group sessions/lectures, small group, and clinical learning center cases	Not addressed	Organ-systems Based	Not addressed	Not addressed	Not addressed	Faculty	
University of South Carolina School of Medicine Greenville^*^	Not applicable	4	Online lectures + hands-on sessions	Flipped classroom	Organ-systems Based	Not addressed	Not addressed	Not addressed	Faculty	5–7: 1
Bangladesh Institute of Health Technology^*^	Not available	2	Lectures, hands-on sessions, observation scanning sessions, field-hospital visits, case presentation	Not addressed	Organ-systems Based	Not addressed	Not addressed	Not addressed	Faculty Sonologist	4: 1
College of Medicine – University of Sharjah^*^	Not applicable	5	Lectures + hands-on sessions	Not addressed	Problem-based learning	Not addressed	Not addressed	Not addressed	Faculty	10: 1
University of Limerick^*^	Not applicable	2	Hands-on annual sessions	Not addressed	Problem-based learning	Not addressed	Not addressed	Not addressed	Faculty Sonographers Visiting POCUS instructors	4–5: 1
Medical College of Georgia School of Medicine^*^	Not available	4	Lectures, hands-on sessions, independent scanning	Not addressed	Other/multi-track	Not addressed	Not addressed	Not addressed	Faculty Peer educators Sonographers	30: 1
University of Texas Health Science Center – San Antonio^*^	Not applicable	4	Didactics + hands-on sessions	Not addressed	Discipline based	Integrated with Clinical Ultrasound Education, clinical rotations, and specialty electives	Not addressed	Not addressed	Faculty Peer educators Fellows and Residents	Variable
Northwestern University Feinberg School of Medicine^*^	Not applicable	4	Hands-on workshops and sessions	Not addressed	Organ-systems based	Not addressed	Hands-on ultrasound workshops during the first two years, SonoSim ultrasound session (knobology and lung)	Optional hands-on sessions for students going into surgical specialties	Faculty	3–5: 1
UNC School of Medicine – Carolinas Medical Center^*^	Lewis et al. [37]	1	Lectures + hands-on sessions	Not addressed	Organ-systems based	Not addressed	Not applicable	Imaging, Acquisition & Orientation The Cardiopulmonary Patient, The Acute Abdomen I, The Acute Abdomen II, The Pregnant Patient, Procedural Guidance, Ultrasound course wrap-up Clerkships	Faculty Residents	4: 1
Rutgers New Jersey Medical School^*^	Not applicable	4	Lectures, online lectures, hands-on sessions	Not addressed	Organ-systems based	Not addressed	Not addressed	Not addressed	Faculty Emergency medicine residents	5: 1
Virginia Commonwealth University^*^	Not applicable	4	Online lectures + hands-on sessions	Not addressed	Organ-systems based	Not addressed	Not addressed	Not addressed	Peer educators	7–8:1
University of California Riverside School of Medicine^*^	Not applicable	4	Didactics + hands-on sessions	Not addressed	Student-driven	Integrated with gross anatomy and clinical skills courses	Gross Anatomy modules Clinical Skills with Point-of-Care Selective	Point-of-care ultrasound sessions	Faculty Peer educators	3: 1
Idaho College of Osteopathic Medicine^*^	Not applicable	4	Didactics, hands-on sessions, podcasts	Not addressed	Organ-systems based	Not addressed	Not addressed	Not addressed	Sonographers	20:1
Texas A&M University^*^	(Texas A&M University, AY 2016–2017)	2	SonoSim, hands-on with SP and ultrasound machine	Not addressed	Longitudinal elective	Ran parallel to clerkships	Not applicable	Fundamentals of Ultrasound Clerkship modules (General Surgery, Emergency Medicine, Critical Care, Family Medicine, Internal Medicine, and OBGYN)	Faculty Peer educators	4–6: 1
Università di Pavia^*^	Not applicable	6	General lectures + small group hands-on sessions	Not addressed	Other/multi-track	Integrated into anatomy and physiology courses	Not addressed	Not addressed	Faculty Peer educators Radiology and Emergency Residents	4: 1

*FAST* focused assessment with sonography for trauma, *FATE* focused assessed transthoracic echocardiography, *OB-GYN* obstetrics and gynecology

#### Duration/curricular structure

Most schools incorporated ultrasound into 1–2 years of UGME but few describe a vertical four-year medical school ultrasound curriculum [1, 5, 8, 9, 12–14]. A majority of undergraduate medical school programs incorporated ultrasound instruction in the pre-clinical years by offering it concurrently with gross anatomy, physical examination, or clinical skills courses or integrating it into similar existing courses in the form of modules. The spectrum of curricular structure was broad at each institution ranging from vertical, organ-systems based, and staged curricula. Consistent training and practice during the clinical years was uncommon. When schools did offer electives or “selectives” in ultrasound, ultrasound education was targeted to a specific specialty that has significant clinical integration of ultrasound (e.g., radiology, obstetrics, emergency ultrasound, etc.) and available to all third- or fourth- year students interested in that specialty.

#### Mode of integration

The mode of integration at institutions varied from: concurrently with anatomy, basic science lectures, and clinical courses; expanded medical program of study; problem-based learning; clinical skills; incorporated into second year practical sessions; integrated into new clinical ultrasound elective, principles of clinical courses, clinical rotations, and specialty electives; and even ran parallel to clerkships.

#### Instructional formats

The format of information delivery across the various programs varied greatly: hands-on sessions, open-access podcast lectures, didactics, web-based learning modules, video assignments, workshops, scanning demonstrations, large and small group sessions, clinical learning through cases, field hospital visit, and independent scanning. A universal characteristic of almost all these programs was delivery of instructional material in a multimodal format, usually as a combination of the methods listed above. Other shared components included the use of the “flipped classroom” instructional method and formal introductory modules for ultrasound imaging and application.

#### Instructors

Programs described varied instructors recruited to teach: faculty, sonographers, specialty-specific residents, and visiting POCUS instructors or specialists. A few ultrasound programs utilized peer educators as the primary instructors for hands-on ultrasound imaging sessions, the rest depended on ultrasound-trained faculty or house-staff with some programs using trained sonographers [8, 15–17]. A few programs included instructor-training sessions to standardize teaching. The student to instructor ratio ranged from 1:2 to 1:30.

#### Evaluation of program

General consensus supports that ultrasound instruction at the UGME level can facilitate efficient patient care and provide a basis for advanced ultrasound training in graduate and continuing medical education [10]. Only a handful of schools reported the impact of ultrasound education UGME in measurable formats like enhanced performance on standardized tests, enhanced physical exam skills by providing real time visual feedback on examination finding, improving the diagnostic accuracy of pathological exam findings, enhancing critical thinking skills by providing real time information corroborating or refuting differential diagnosis [5, 18–21]. One school conducted a retrospective analysis of the Comprehensive Osteopathic Medical Licensing Examination of the United States (COMLEX-USA) level 1 scores in anatomy, diagnostic technology, and osteopathic principals and practice. Scores markedly increased across all three areas from twenty or more below the national mean to up to twenty-eight points above the national mean over three years attributable to ultrasound integration.

## Integrated ultrasound curriculum development at wake forest school of medicine

### Curricular structure prior to implementation of ultrasound curriculum

**Fig. 2:**
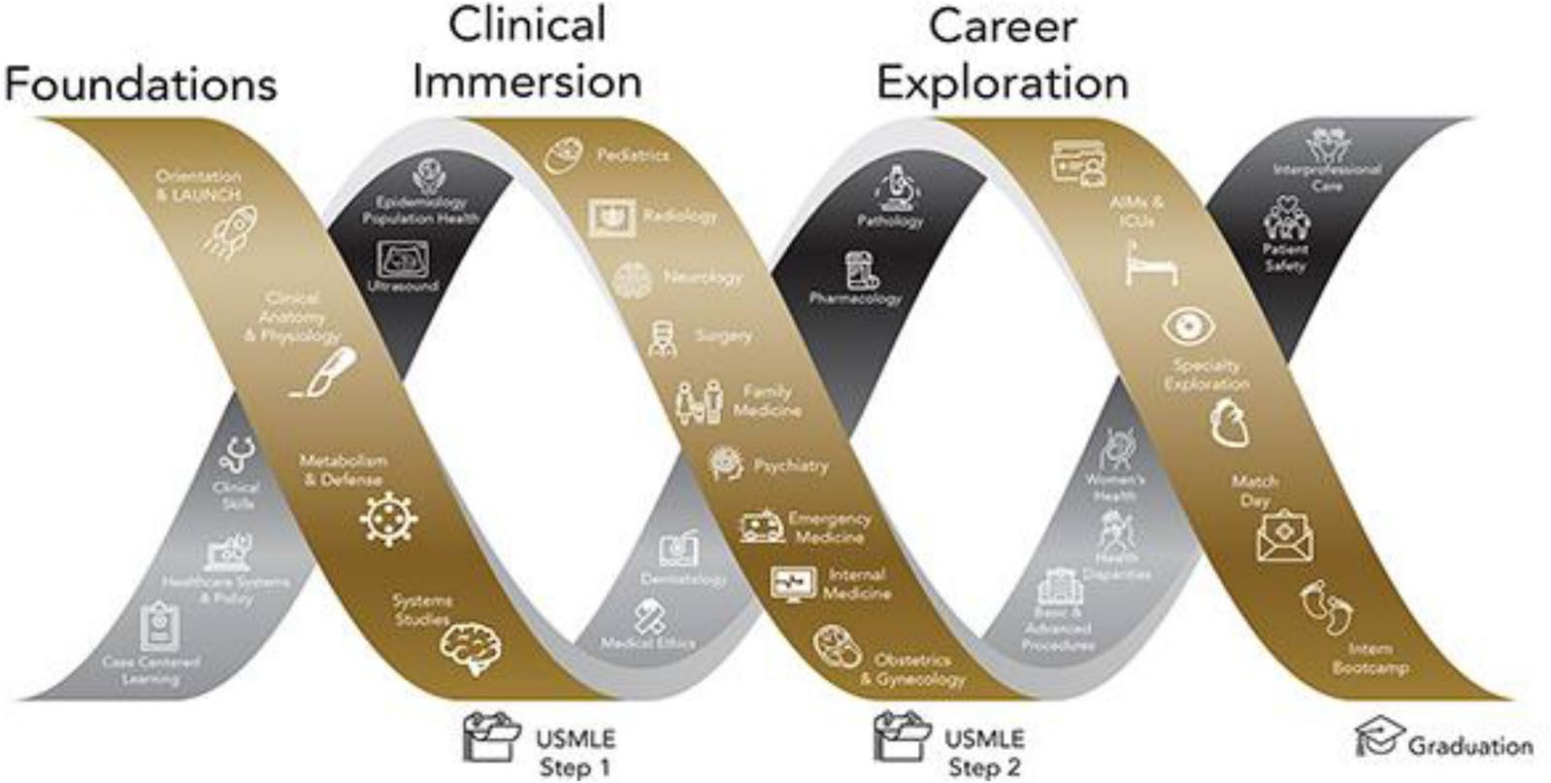
The Wake Ready! Curriculum is divided into three phases to better prepare medical students for the next step in their training. Phases are divided into requisite focuses as Foundations, Clinical Immersion and Career Exploration

Like most Liaison Committee on Medical Education (LCME) accredited programs, Wake Forest institutional curriculum for UGME Doctor of Medicine (MD) students consists of 18 months of pre-clinical courses in human anatomy, histology, bioethics, population health, and systems-based pathophysiology in a variety of instructional formats including lectures, laboratory experiences, small group exercises, case-based learning, and asynchronous education. Students transition to clinical rotations in February of their second academic year and begin senior acting internship and elective experiences in March of their third academic year. Major components of the curriculum are delivered in a block format where topics follow each other through the calendar year (Fig. 2). The MD UGME program includes non-block format “threads” which are taught, to 150 students per year, in tandem with other curricular elements throughout all 4 years. Examples at our institution include pharmacology, bioethics, population health, physical exam and diagnosis (Tables 3 and 4). Threads are taught in parallel to the underlying block schedule to emphasize the thread content most relevant to the current pathophysiology block. We describe the evolution of this curriculum based on the 6-step framework provided by Kern for curricular development in medical education [4].

- Problem identification and general needs assessment: An institutional needs analysis demonstrated fading command of anatomy and physiology concepts after the pre-clinical years that was not being addressed with current curricular structure. In exploring solutions, the Departments of Emergency Medicine and Neurology were found to have existing educational models integrating ultrasound as a visual and interactive tool to reinforce clinical concepts with access to ultrasound machines and ultrasound-trained faculty.
- Targeted needs assessment: After prioritizing the need to develop an integrated ultrasound curriculum, an approach of problem-centered curriculum design was chosen and a team of faculty with content expertise was created. Town hall and in-person meetings with UGME course directors and clinical leaders were held to identify the content that would gain the most from hands-on ultrasound experiences to target synergy between ultrasound laboratory exercises and the objectives of the general medical student curriculum.
- Goals and objectives: Frequency and duration of these ultrasound didactic modules and labs was designed to focus on the use of ultrasound as a “clinical tool” to teach pre-clinical students anatomy in Year 1 and physiology in Year 2 with minimum course scheduling changes in existing structure.
- Educational strategies and evaluation/feedback: Above efforts led to integration of hands-on ultrasound as one of these longitudinal threads in UGME in 2014 to provide an enhanced learning experience for medical students. For each component of the curriculum, the pedagogy that seemed most promising to address the problem of fading basic science concepts among students and application in clinical years and is described below specific to each year.
  i. Year 1 and 2 preclinical curriculum: The curriculum was conceptualized in a “flipped classroom” model to include didactic instruction through online modules followed by laboratory component with hands-on ultrasound training labs with greater emphasis on experiential learning (Tables 3 and 4) [6]. Online modules were recorded with easily available software (QuickTime, Apple, Inc., 1991) as 20–60 min online screencast lectures posted on the medical school learning management system (Canvas, Instructure, 2011). Didactic content emphasizes recognition of normal anatomic relationships between organs and tissues using ultrasound cine loops with information on ultrasound scanning technique, image interpretations and clinical applications. Student assessment is performed via formative pre-tests and summative post-testing questions. Ultrasound hands-on lab sessions occur over 4-h blocks. The 1-h session is repeated hourly for a quarter of the class (30–40 students) per session. Students are divided into groups of 4–5 students per machine and facilitated by one to four faculty members per session. Lab sessions move methodically through a series of imaging objectives focusing on scanning techniques and anatomy identification. The remaining students not participating in ultrasound lab are often in other parallel class activities such as anatomy dissection or small group sessions.
    Student assessment is performed via formative pre-tests and summative post-testing questions that are course specific quizzes. We also incorporated ultrasound related content on core curricular exams. Lab participation is mandatory and forms a component of the course grade. For each module, students complete a formative pre-test quiz assessing their general ultrasound knowledge and content specific anatomic knowledge before reviewing the online lecture that is followed by another summative quiz testing their grasp on image interpretation in relation to anatomy, physiology and clinical concepts.
  ii. Year 3 clinical curriculum: Increasing popularity and positive feedback from medical students participating in the curriculum resulted in extending the ultrasound component to the UGME clinical clerkship year in 2016. The didactic and lab sessions follow the similar flipped classroom model with content focused on clinical concepts corresponding to the clinical clerkship. After an online review of lectures reorienting students to relevant ultrasound anatomy and physiology, one-hour sessions are organized by physicians during the clinical clerkships under the guidance of the ultrasound curriculum leadership (Table 4). The sessions are designed to focus on structured bedside exams commonly used by providers in the patient care environment. In rotations like obstetrics, ultrasound was focused on pregnancy and fetal pig phantoms were used to highlight clinical concepts [7]. Following formal didactic and laboratory sessions, students are encouraged to participate in bedside ultrasound exams as opportunities arise during their clinical clerkship.
  iii. Year 4 clinical curriculum: The final year of ultrasound curriculum for year 4 was added in 2017 as a POCUS elective unique to a handful of programs [8]. This multidisciplinary elective was initially offered to 1 student per block and now accommodates 2 students every block and taught 22 students in its third year. The focus of this 4-week elective is hands-on experience designed to improve bedside image acquisition and skills in point-of-care examinations. Student rotate in emergency and critical care settings in the first two weeks and subspecialty experiences in the final two weeks to increase the awareness of clinical applications of ultrasound in diagnostic and point-of-care use across various subspecialties. Elective rotation settings include: Emergency Medicine, Neurology, Medical-Critical Care, Neurocritical Care, Cardiology, Obstetrics and Gynecology (OB/GYN), Trauma Surgery, Vascular Surgery, Pediatric Cardiology, Internal Medicine, Community Medicine, and Sports Medicine. Students maintain a digital portfolio of at least 25 scans during this rotation [9]. The POCUS elective is a very distinctive and popular component of Wake Forest’s UGME curriculum due to its multidisciplinary format packaged within one block translating bedside imaging into clinical concepts across a wide range of specialties. Assessment for the 4th year elective occurs through direct observation of scanning and clinical application concepts, review of case logs, online quizzes, a mandatory student led presentation or review of ultrasound-related research article on an ultrasound topic of their choice.

**Table 3:** First and second year medical student ultrasound curriculum as part of a longitudinal program at wake forest

MS 1 Ultrasound curriculum				
Course learning objective	Method of assessment (if applicable):	Institutional objective domain	Institutional objective subdomains	Entrustable professional activities (EPA)
1. Anatomy I—Intro to Ultrasound: Describe the basic scientific principles of diagnostic ultrasound Describe the basic principles of ultrasound image acquisition Identify the median nerve, tendons of the forearm muscles, and the carpal tunnel	AM09: Multisource Assessment AM12: Participation AM17: Self-Assessment AM19: Exam – Institutionally Developed, Laboratory, Practical	1.Knowledge for Practice 2. Interpersonal and Communication Skills 3. Patient Care 4. Professionalism	1.0 1.1 2.0 2.4 3.0 3.4 4.0	EPA 1 EPA 3 EPA 10 EPA 12
2. Anatomy II—The Heart: Identify the major chambers of the heart Identify the aortic, tricuspid, and mitral valves Correlate the heart sounds with valve motions	AM09: Multisource Assessment AM12: Participation AM17: Self-Assessment AM19: Exam–Institutionally Developed, Laboratory, Practical	1. Knowledge for Practice 2. Interpersonal and Communication Skills 3. Patient Care 4. Professionalism	1.0 1.1 2.0 2.4 3.0 3.4 4.0	EPA 1 EPA 3 EPA 10 EPA 12
3. Anatomy III – The Abdomen and Pelvis: Obtain images of and identify the liver, spleen and kidneys in two anatomic planes (1.0, 1.1) Identify the major potential spaces in the abdomen (1.0. 1.1) Image and identify the uterus, prostate, bladder (1.0, 1.1) Identify the pelvic potential space (1.0, 1.1)	AM09: Multisource Assessment AM12: Participation AM17: Self-Assessment AM19: Exam – Institutionally Developed, Laboratory, Practical	1. Knowledge for Practice 2. Interpersonal and Communication Skills 3. Patient Care 4.Professionalism	1.0 1.1 2.0 2.4 3.0 3.4 4.0	EPA 1 EPA 3 EPA 10 EPA 12
4. Anatomy IV – The Neck: Identify the trachea, thyroid gland, common carotid artery, and internal jugular vein Describe the characteristics of simple cysts	AM09: Multisource Assessment AM12: Participation AM17: Self-Assessment AM19: Exam–Institutionally Developed, Laboratory, Practical	1. Knowledge for Practice 2. Interpersonal and Communication Skills 3. Patient Care 4. Professionalism	1.0 1.1 2.0 2.4 3.0 3.4 4.0	EPA 1 EPA 3 EPA 10 EPA 12
5. Anatomy V – Musculoskeletal/Shoulder: Identify the biceps tendon Describe the sonographic appearance of muscle, tendon, and bone Identify the major components of the rotator cuff	AM09: Multisource Assessment AM12: Participation AM17: Self-Assessment AM19: Exam–Institutionally Developed, Laboratory, Practical	1. Knowledge for practice 2. Interpersonal and communication skills 3. Patient care 4. Professionalism	1.0 1.1 2.0 2.4 3.0 3.4 4.0	EPA 1 EPA 3 EPA 10 EPA 12
6. Neuroscience–Carotid: Perform color Doppler imaging of common carotid flow. (1.0, 1.1) Brain: Image the MCA with transcranial Doppler techniques. (1.0, 1.1)	AM09: Multisource Assessment AM12: Participation AM17: Self-Assessment AM19: Exam – Institutionally Developed, Laboratory, Practical	1. Knowledge for practice 2. Interpersonal and communication skills 3. Patient care 4. Professionalism	1.0 1.1 2.0 2.4 3.0 3.4 4.0	EPA 1 EPA 3 EPA 10 EPA 12
7. Gastroenterology—Biliary Anatomy: Assess liver span and correlate with palpation of the liver edge (1.0, 1.1) Identify the portal vein and vena cava (1.0, 1.1) Measure the size of the gallbladder (1.0, 1.1) Identify the common bile duct (1.0, 1.1)	AM09: Multisource Assessment AM12: Participation AM17: Self-Assessment AM19: Exam–Institutionally Developed, Laboratory, Practical	1. Knowledge for practice 2. Interpersonal and communication skills 3. Patient care 4. Professionalism	1.0 1.1 2.0 2.4 3.0 3.4 4.0	EPA 1 EPA 3 EPA 10 EPA 12
8. Pulmonology – Lung: Assess lung slide (1.0, 1.1) Assess diaphragm – excursion (1.0, 1.1) Identify diaphragm in ultrasound image (1.0, 1.1)	AM09: Multisource Assessment AM12: Participation AM17: Self-Assessment AM19: Exam–Institutionally Developed, Laboratory, Practical	1. Knowledge for Practice 2. Interpersonal and communication skills 3. Patient care 4. Professionalism	1.0 1.1 2.0 2.4 3.0 3.4 4.0	EPA 1 EPA 3 EPA 10 EPA 12
9. Cardiopulmonary – The Heart I Identify and Assess mitral valve motion (1.0, 1.1) Identify diastole and systole on echocardiographic images (1.0, 1.1) Associate heart sounds with cardiac motion (1.0, 1.1)	AM09: Multisource Assessment AM12: Participation AM17: Self-Assessment AM19: Exam – Institutionally Developed, Laboratory, Practical	1. Knowledge for practice 2. Interpersonal and communication skills 3. Patient care 4. Professionalism	1.0 1.1 2.0 2.4 3.0 3.4 4.0	EPA 1 EPA 3 EPA 10 EPA 12
10. Cardiopulmonary – Cardiac Functional Assessment: Measure the E-point septal separation (1.0, 1.1) Measure the ejection fraction in the LV 2 chamber view and the Apical 4 chamber view (1.0, 1.1)	AM09: Multisource Assessment AM12: Participation AM17: Self-Assessment AM19: Exam – Institutionally Developed, Laboratory, Practical	1. Knowledge for practice 2. Interpersonal and communication skills 3. Patient care 4. Professionalism	1.0 1.1 2.0 2.4 3.0 3.4 4.0	EPA 1 EPA 3 EPA 10 EPA 12
11. Renal – The urinary tract: Identify the right and left kidneys (1.0, 1.1) Measure the size of a kidney (1.0, 1.1) Measure the volume of the bladder (1.0, 1.1) Identify ureteral jets in the bladder (1.0, 1.1)	AM09: Multisource Assessment AM12: Participation AM17: Self-Assessment AM19: Exam – Institutionally Developed, Laboratory, Practical	1. Knowledge for practice 2. Interpersonal and communication skills 3. Patient care 4. Professionalism	1.0 1.1 2.0 2.4 3.0 3.4 4.0	EPA 1 EPA 3 EPA 10 EPA 12
12. Endo—Thyroid: Identify the thyroid gland (1.0, 1.1) Identify the trachea (1.0, 1.1) Measure the volume of the thyroid gland (1.0, 1.1)	AM09: multisource assessment AM12: Participation AM17: Self-Assessment AM19: Exam – Institutionally Developed, Laboratory, Practical	1. Knowledge for Practice 2. Interpersonal and communication skills 3. Patient care 4. Professionalism	1.0 1.1 2.0 2.4 3.0 3.4 4.0	EPA 1 EPA 3 EPA10 EPA 13

* MS1 * first year medical student,* EPA* entrustable professional activities, *MCA* middle cerebral artery,* LV * left ventricular

**Table 4:** Third year medical student ultrasound curriculum as part of a longitudinal program at wake forest

Exam	Rotation	Learning Objectives
Bedside echo	Internal medicine	1. Describe indications for a bedside echocardiogram 2. Describe ultrasound findings related to intravascular volume status 3. Describe the sonographic characteristics of tamponade on 2D imaging 4. Describe ultrasound findings related to systolic dysfunction 5. Demonstrate the standard echocardiographic views for the exam
Focused assessment with sonography in trauma	Surgery	1. Describe indications for a FAST exam 2. Describe implications for downstream patient care based on exam findings 3. Describe the appearance of abdominal free fluid in each sonographic window 4. Describe the sonographic characteristics of tamponade on 2D imaging 5. Demonstrate the standard ultrasound views for the exam
Ultrasound in pregnancy	Obstetrics and gynecology	1. Describe the indications for obstetric ultrasound 2. Optimize quality of fetal images 3. Determine fetal lie and presentation 4. Identify the fetal heads, spine, extremities, abdomen, and thorax 5. Visualize anatomic structures in more than one orthogonal plane
Ultrasound guided procedures	Emergency medicine	1. Describe the differences between in plane and out of plane needle guidance 2. Describe general principles of ultrasound guided procedures 3. Describe the procedural steps for an ultrasound guided IV catheter placement 4. Perform in plane and out of plane needle guidance on an ultrasound phantom

*FAST* focused assessment with sonography for trauma, *2D* two-dimensional, *IV* intravenous

### Implementation

A private philanthropic grant awarded by The Fullerton Foundation in 2014 totaling $300,000 was secured to fund this new model for 3 years. The grant was earmarked for faculty effort to design curriculum; collaboration effort from University of South Carolina faculty as we developed curriculum; program staff support; travel to conferences; and capital equipment including 4 machines and consumable supplies. Matching institutional funds provided seed money for faculty release time to develop education modules and deliver hands-on sessions for medical students. Because the University of South Carolina School of Medicine–Greenville (USC-G) was one of the first institutions with an integrated curriculum, we sought out a collaboration with faculty at USC-G to ensure a streamlined launch of our UGME curriculum [1, 3]. Loaned equipment from the Center for Medical Ultrasound and Department of Emergency Medicine supplemented the machines purchased from seed grant. Program infrastructure detail are described in Table 5.

**Table 5:** Infrastructural details of program development at wake forest school of medicine

Machines	Initial grant funding 4 cart-based machines, each with a phased array, curvilinear and linear probe One owned by the institution machine on permanent loan from the manufacturer The success of the curriculum allowed additional machine purchases possible, to a total of 9 cart-based machines, each with a 3-probe setup
Physical space requirements	A room large enough for 1 to 3 instructors to supervise 5–6 groups of students Stretchers or exam tables are needed so that students or standardized models can lie recumbent for exams The lab sessions initially were held in an available large meeting room in the medical school building. In 2017 we moved to a new building and held ultrasound laboratory sessions in a large multipurpose room
Audiovisual	We have found it is also very helpful to have large secondary displays for group members to observe that can be repositioned so that student models can also see the image At curriculum launch, we purchased gurneys for use during ultrasound lab sessions and two large LCD displays on portable mounts 2017 converted to with 5 mounted displays as well as our exiting mobile displays
Personnel requirements	Faculty/sonographer hours per session 4–16 h (yearly estimate)

*LCD* liquid–crystal display

**Fig. 3:**
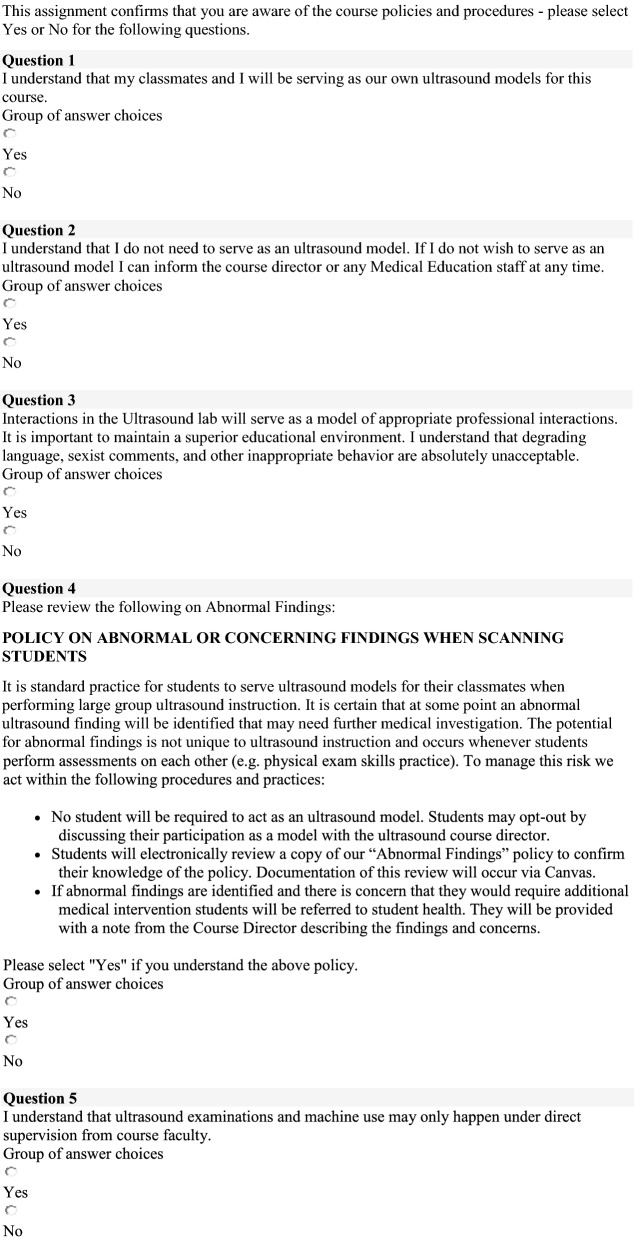
Wake Forest School of Medicine Student Model Policy and Policy for Management of Incidental Findings

In all lab sessions through pre-clinical and clinical year, students volunteer as ultrasound models for each other and perform ultrasound imaging of relevant anatomic structures for each module over their assigned lab. Participation as an ultrasound model is voluntary with a high rate of participation and yearly electronic acknowledgment of the Student Model Policy and Policy for Management of Incidental Findings (Fig. 3). Students with incidental findings are referred to our student health system with initial direct communication between the ultrasound course director, the student, and the director of student health. To date, an annual average of 5 of 360 students over 31 hours of labs scanning have been referred for additional evaluation of incidental findings. Laboratory sessions associated with an increased risk of referral include Neck and Endocrine Laboratory sessions (2 students with thyroid nodules or mass) and Abdomen/Renal sessions (2 students with unilateral renal agenesis, one student with splenic lesion).

## Discussion

The last 10 years have seen UGME instruction in hands-on ultrasound skills go from niche to mainstream. Institutions opt to integrate ultrasound education into their medical school curriculum primarily because ultrasonography offers the potential to be an efficient educational tool that enhances traditional learning of anatomy, medical physiology, and clinical skills [5, 8, 14, 16]. Published programs have demonstrated the feasibility of integrating a POCUS curriculum into UGME and emerging data suggests that students perceive value in this effort. Focused ultrasonography can be used as a useful aid in teaching anatomy to medical students, learning physical exam skills; and has been received quite positively by medical students at several institutions [8, 21]. A national survey of 82 medical schools, where 51 report some ultrasound integration into UGME, found that > 90% of students preferred ultrasound incorporated into their program, > 80% felt that ultrasound sessions enhanced training in both pre-clinical and clinical courses, and 60–90% students reported increased confidence in physical exam skills after visualizing anatomy by ultrasound [1, 5, 11, 14, 21]. In 2014, fewer than 60% of medical schools reported some level of ultrasound training in UGME; and almost 80% agreed that it should be included in UGME but fewer than 20% prioritized it [22]. The American Institute of Ultrasound in Medicine hosts a self-reported list of all medical schools with UGME ultrasound curricula and reports 66 of 222 LCME accredited schools have some level of structured ultrasound instruction. Only 25 list a 4-year ultrasound curriculum [1, 5, 8, 12, 23, 24]. Though national consensus curricula have been published by several organizations, lack of guidelines or LCME inclusion of ultrasound similar to the Accreditation Council for Graduate Medical Education (ACGME) endorsement for emergency medicine residency is a barrier towards widespread POCUS UGME programs [25–27].

The primary challenge for institutions initiating such a curriculum is funding the required equipment and the dedicated faculty instructor time. Prior published ultrasound curricula relied on industry funding at startup, which is not a consistent or reliable resource [1, 5]. Integration of ultrasound into UGME also requires trained faculty well-versed in indications, limitations, benefits, and risks of POCUS; protecting their effort to dedicate to ultrasound education is a challenge [2]. We were fortunate to receive a private grant to fund the initial infrastructure set up that made matching institutional funds available to expand and continue the curriculum in its current form. We attempted to address faculty costs by adding a dedicated sonographer and peer-to-peer education by senior medical students. Additional challenges for institutions initiating such a curriculum is administrative buy-in and finding time to implement this curriculum into an already stacked curriculum.

Over the past few years, the ultrasound curriculum at WFSOM has experienced tremendous growth. Our curriculum has expanded from 8 modules in 2014 to 16 modules in 2020 with an additional ultrasound elective equaling 100 contact hours (Table 1). An active ultrasound interest group initiated by students collaborates with our faculty to develop extracurricular lab sessions on topics of interest that sometimes address ongoing innovation in subspecialty ultrasound clinical application or technology [11]. An ultrasound certificate program was introduced in 2019 to encourage clinical and research activities in ultrasound. Students choose a precepted ultrasound related activity ranging from a research project with a faculty mentor to a defined number of sessions serving as facilitators for pre-clinical medical students. Ultrasound interest group activities also contribute to certificate requirements.

The ultrasound curriculum at WFSOM shares prominent themes and characteristics with other ultrasound curricula across different institutions yet offers its own unique opportunities. Our curriculum is integrated across all four years of UGME, is structured to complement organ system-based blocks, and is formatted to present information in a multimodal fashion through a combination of lectures and hands-on lab sessions. Despite emphasis on anatomy, physiology and clinical concepts, the degree of exposures lends itself to significant knowledge in ultrasound image acquisition and interpretation. At WFSOM, administrative buy-in was relatively simple. There was an identified priority to provide our medical students with additional learning experiences in the areas of anatomy and physiology as well as important clinical experiences to reinforce learning. We fashioned dedicated time for ultrasound by leveraging co-existing content on the UGME academic calendar. Formatting lectures as online on-demand with a “flipped classroom” model also helped to keep the footprint within the school day as small as possible. One of our more distinctive features, the curriculum offers opportunities for specialty-specific ultrasound training during certain clerkship rotations integrated into teaching bedside examinations, the POCUS elective offered to fourth-year students, and the option to participate in the ultrasound certificate program.

## Conclusion

Ultrasound integration can be a useful assistive educational method in undergraduate medical education but limited by resource intensive set up and high variability of program integration into UGME curricula. Common standards, milestones, and standardized competency-based assessments would be helpful in more application that is widespread.

## Data Availability

Data sharing is not applicable to this article as no datasets were generated or analyzed during the current study.

## Appendix

See Table 6.

**Table 6:** Ultrasound Curricula in Medical Education Survey

1. Is your institution’s ultrasound curriculum published?
Yes
No
2. If possible, please provide full-text reference
3. Ultrasound education at your institution is integrated into:
a. 1 year of the medical school curriculum
b. 2 years of the medical school curriculum
c. 3 years of the medical school curriculum
d. All 4 years of the medical school curriculum
4. What is the format of information delivery (i.e., lectures, hands-on sessions, etc.)?
5. Instructors are primarily (Check all that apply):
a. Faculty
b. Peer educators–senior medical students
c. Sonographer
d. All the above
e. Other (please specify)
6. .What is the student-to-instructor ratio for hands-on sessions?
7. Please let us know if there’s anything that’s unique to your program that you think others may not offer
8. Please enter your name and associated email address to help us link responses to institutions

See [35]

## Abbreviations

ACGME: Accreditation Council for Graduate Medical Education
COMLEX-USA: Comprehensive Osteopathic Medical Licensing Examination of the United States
EPA: Entrustable professional activities
LCME: The Liaison Committee on Medical Education
MD: Doctor of Medicine
OB/GYN: Obstetrics and gynecology
POCUS: Point-of-care ultrasound
PRISMA: Preferred Reporting Items for Systematic Reviews and Meta-Analyses
UGME: Undergraduate medical education
USC-G: University of South Carolina School of Medicine–Greenville
US: United States
WFSOM: Wake Forest School of Medicine

## References

[1] Hoppmann RA, Rao VV, Bell F (2015). The evolution of an integrated ultrasound curriculum (iUSC) for medical students: 9-year experience. Crit Ultrasound J.

[2] Tarique U, Tang B, Singh M, Kulasegaram KM, Ailon J (2018). Ultrasound curricula in undergraduate medical education: a scoping review. J Ultrasound Med.

[3] Tricco AC, Lillie E, Zarin W (2018). PRISMA extension for scoping reviews (PRISMA-ScR): checklist and explanation. Ann Intern Med.

[4] Kern DE, Thomas PA, Hughes MT (2009). Curriculum development for medical education: six-step approach.

[5] Hoppmann RA, Rao VV, Poston MB (2011). An integrated ultrasound curriculum (iUSC) for medical students: 4-year experience. Crit Ultrasound J.

[6] Shokoohi H, Boniface K, Kaviany P, Armstrong P, Calabrese K, Pourmand A (2016). An experiential learning model facilitates learning of bedside ultrasound by preclinical medical students. J Surg Educ.

[7] Akoma UN, Shumard KM, Street L, Brost BC, Nitsche JF (2015). Impact of an inexpensive anatomy-based fetal pig simulator on obstetric ultrasound training. J Ultrasound Med.

[8] Bahner DP, Royall NA (2013). Advanced ultrasound training for fourth-year medical students: a novel training program at The Ohio State University College of Medicine. Acad Med.

[9] Hughes DR, Kube E, Gable BD, Madore FE, Bahner DP (2012). The sonographic digital portfolio: a longitudinal ultrasound image tracking program. Crit Ultrasound J.

[10] Davis JJ, Wessner CE, Potts J, Au AK, Pohl CA, Fields JM (2018). Ultrasonography in Undergraduate Medical Education: A Systematic Review. J Ultrasound Med.

[11] Chiem AT, Soucy Z, Dinh VA (2016). Integration of ultrasound in undergraduate medical education at the California medical schools. J Ultrasound Med.

[12] Bahner DP, Adkins EJ, Hughes D, Barrie M, Boulger CT, Royall NA (2013). Integrated medical school ultrasound: development of an ultrasound vertical curriculum. Crit Ultrasound J.

[13] Rao S, van Holsbeeck L, Musial JL (2008). A pilot study of comprehensive ultrasound education at the Wayne State University School of Medicine: a pioneer year review. J Ultrasound Med.

[14] Afonso N, Amponsah D, Yang J (2010). Adding new tools to the black bag–introduction of ultrasound into the physical diagnosis course. J Gen Intern Med.

[15] Rempell JS, Saldana F, DiSalvo D (2016). Pilot point-of-care ultrasound curriculum at Harvard Medical School: early experience. West J Emerg Med.

[16] Knobe M, Munker R, Sellei RM (2010). Peer teaching: a randomised controlled trial using student-teachers to teach musculoskeletal ultrasound. Med Educ.

[17] Celebi N, Griewatz J, Malek NP (2019). Development and implementation of a comprehensive ultrasound curriculum for undergraduate medical students - a feasibility study. BMC Med Educ.

[18] Shapiro RS, Ko PK, Jacobson S (2002). A pilot project to study the use of ultrasonography for teaching physical examination to medical students. Comput Biol Med.

[19] Amini R, Stolz LA, Hernandez NC (2016). Sonography and hypotension: a change to critical problem solving in undergraduate medical education. Adv Med Educ Pract.

[20] Stokke TM, Ruddox V, Sarvari SI, Otterstad JE, Aune E, Edvardsen T (2014). Brief group training of medical students in focused cardiac ultrasound may improve diagnostic accuracy of physical examination. J Am Soc Echocardiogr.

[21] Zawadka M, Graczynska A, Janiszewska A (2019). Lessons learned from a study of the integration of a point-of-care ultrasound course into the undergraduate medical school curriculum. Med Sci Monit.

[22] Bahner DP, Goldman E, Way D, Royall NA, Liu YT (2014). The state of ultrasound education in U.S. medical schools: results of a national survey. Acad Med.

[23] Medicine AIoUi. Ultrasound in Medical Education Portal http://meded.aium.org/medical-schools. Accessed March 19, 2020.

[24] Minardi J, Ressetar H, Foreman T (2019). Longitudinal ultrasound curriculum incorporation at West Virginia University School of Medicine: a description and graduating students' perceptions. J Ultrasound Med.

[25] Baltarowich OH, Di Salvo DN, Scoutt LM (2014). National ultrasound curriculum for medical students. Ultrasound Q.

[26] Recommended Curriculum Guidelines for Family Medicine Residents-Point of Care Ultrasound. https://www.aafp.org/dam/AAFP/documents/medical_education_residency/program_directors/Reprint290D_POCUS.pdf. Published 2020. Accessed March 19, 2020.

[27] National Competence Based Catalogue of Learning Objectives for Undergraduate Medical Education (NKLM). http://www.nklm.de/files/nklm_final_2015-07-03.pdf. Published 2015. Accessed March 19, 2020.

[28] Hendriksz T, Markman Z, Pera A (2018). An education in osteopathic ultrasonography (AEIOU) program: one institution's approach to advancing an ultrasonography curriculum. J Am Osteopath Assoc.

[29] Fox JC, Schlang JR, Maldonado G, Lotfipour S, Clayman RV (2014). Proactive medicine: the "UCI 30," an ultrasound-based clinical initiative from the University of California. Irvine Acad Med.

[30] Wilson SP, Mefford JM, Lahham S (2017). Implementation of a 4-Year point-of-care ultrasound curriculum in a liaison committee on medical education-accredited US medical school. J Ultrasound Med.

[31] Nelson BP, Hojsak J, Dei Rossi E, Karani R, Narula J (2017). Seeing is believing: evaluating a point-of-care ultrasound curriculum for 1st-year medical students. Teach Learn Med.

[32] Moscova M, Bryce DA, Sindhusake D, Young N (2015). Integration of medical imaging including ultrasound into a new clinical anatomy curriculum. Anat Sci Educ.

[33] Kondrashova T, Lockwood MD (2015). Innovative approach to teaching osteopathic manipulative medicine: the integration of ultrasonography. J Am Osteopath Assoc.

[34] Kondrashova T, Kondrashov P (2018). Integration of ultrasonography into the undergraduate medical curriculum: seven years of experience. Mo Med.

[35] Thomas PA, Kern DE, Hughes MT, Chen BY (2015). Curriculum development for medical education: a six-step approach.

[36] Russ BA, Evans D, Morrad D, Champney C, Woodworth AM, Thaut L, Thiessen M (2017). Integrating point-of-care ultrasonography into the osteopathic medical school curriculum. J Am Osteopath Assoc..

[37] Lewis MR, Howley L, White P, Colcord C, Allen BK (2018). Development and evaluation of a longitudinal integrated ultrasound curriculum for third yearmedical students. J Reg Med Campuses..

